# Adiabatic quantum linear regression

**DOI:** 10.1038/s41598-021-01445-6

**Published:** 2021-11-09

**Authors:** Prasanna Date, Thomas Potok

**Affiliations:** Computer Science and Mathematics Division, Oak Ridge National Laboratory, Oak Ridge, 37830 USA

**Keywords:** Computer science, Quantum information, Statistics

## Abstract

A major challenge in machine learning is the computational expense of training these models. Model training can be viewed as a form of optimization used to fit a machine learning model to a set of data, which can take up significant amount of time on classical computers. Adiabatic quantum computers have been shown to excel at solving optimization problems, and therefore, we believe, present a promising alternative to improve machine learning training times. In this paper, we present an adiabatic quantum computing approach for training a linear regression model. In order to do this, we formulate the regression problem as a quadratic unconstrained binary optimization (QUBO) problem. We analyze our quantum approach theoretically, test it on the D-Wave adiabatic quantum computer and compare its performance to a classical approach that uses the Scikit-learn library in Python. Our analysis shows that the quantum approach attains up to ${2.8 \times }$ speedup over the classical approach on larger datasets, and performs at par with the classical approach on the regression error metric. The quantum approach used the D-Wave 2000Q adiabatic quantum computer, whereas the classical approach used a desktop workstation with an 8-core Intel i9 processor. As such, the results obtained in this work must be interpreted within the context of the specific hardware and software implementations of these machines.

## Introduction

Machine learning algorithms and applications are ubiquitous in our day-to-day lives and are deployed on a variety of devices—from edge devices like smartphones to large supercomputers. Before they are deployed in a real world application, machine learning models need to be trained, which is a time intensive process, and can even take a few months. When training machine learning models, we usually minimize a well-defined error function using state-of-the-art optimization techniques such as gradient descent, ellipsoid method and evolutionary optimization^1^.

While seemingly efficient on smaller problems, these optimization techniques tend to become infeasible as the problem size grows despite the polynomial time complexity. The reasons for this stem from the implementation-specific details at the hardware and software level. Prominent issues include increased communication cost nullifying the gains in the computation cost as the problem size increases, difficulty in managing finite compute and memory resources, and algorithms optimized for small-to-mid-sized problems^2–4^. In this light, and given that the Moore’s law is nearing its inevitable end, it is necessary to explore the applicability of non-conventional computing paradigms like quantum computing for solving large-sized optimization problems, including training machine learning models.

Quantum computers are known to be good at solving hard optimization problems and offer a promising alternative to accelerate the training of machine learning models^5^. For instance, adiabatic quantum computers like the D-Wave 2000Q can approximately solve NP-complete problems like the quadratic unconstrained binary optimization (QUBO) problem, and have been used to train machine learning models like Restricted Boltzmann Machines (RBMs) and Deep Belief Networks (DBNs) in classical-quantum hybrid approaches^6^. Although today’s quantum computers are small, error-prone and in the noisy intermediate-scale quantum (NISQ) era, the future machines are sought to be large, reliable and scalable^7,8^.

In this paper, we evaluate the use of adiabatic quantum computers to train linear regression models. Linear regression is a machine learning technique that models the relationship between a scalar dependent variable and one or more independent variables^9^. It has applications in business, economics, astronomy, scientific analysis, weather forecasting, risk analysis etc.^10–15^. It is not only used for prediction and forecasting, but also to determine the relative importance of data features. Linear regression has an analytical solution and can be solved in ${\mathscr {O}}(N^3)$ time on classical computers, where *N* is the size of the training data.

The main contributions of this work are as follows:

1. We propose a quantum approach to solve the linear regression problem by formulating it as a quadratic unconstrained binary optimization (QUBO) problem.
2. We theoretically analyze our quantum approach and demonstrate that its run time is equivalent to that of current classical approaches.
3. We empirically test our quantum approach using the D-Wave 2000Q adiabatic quantum computer and compare its performance to a classical approach that uses the Scikit-learn library in Python. The performance metrics used for this comparison are regression error and computation time. We show that both approaches achieve comparable regression error, and that the quantum approach achieves $2.8\times$ speedup over the classical approach on larger datasets.

## Related work

Linear regression is one of the most widely used statistical machine learning techniques. Bloomfield and Steiger propose a method for least absolute deviation curve fitting, which was three times faster than the ordinary least squares approach^16^. Megiddo and Tamir propose ${\mathscr {O}}(N^2 \log N)$ and ${\mathscr {O}}(N \log ^2 N)$ algorithms for regression based on the Euclidean error and the rectilinear ($l_1$) error respectively, where *N* is the number of datapoints in the training dataset^17^. Zemel propose ${\mathscr {O}}(N)$ algorithm for linear multiple choice knapsack problem, which translates to linear regression with rectilinear error^18^.

Theoretically, the best classical algorithm for linear regression, has time complexity ${\mathscr {O}}(N d^{1.37})$ using a fast matrix multiplication algorithm, such as Coppersmith–Winograd^19^, where *N* is the number of data points in the training data set and *d* is the number of features. However, most practical implementations in widely used machine learning libraries like the Scikit-learn library in Python run in ${\mathscr {O}}(N d^2)$ time^20,21^. ${\mathscr {O}}(N d^2)$ appears to be the most widely accepted time complexity for linear regression, and will be the basis of comparison in this paper.

Quantum algorithms have also been explored for linear regression in the literature. Harrow et al. propose a quantum algorithm for solving a system of linear equations, that runs in $\texttt {poly}(\log N, \kappa )$ time, where $\kappa$ is the condition number of the input matrix^22^. Schuld et al. propose an algorithm for linear regression with least squares that runs in logarithmic time in the dimension of input space provided training data is encoded as quantum information^23^. Wang proposes a quantum linear regression algorithm that runs in $\texttt {poly}(\log _2 N, d, \kappa , \frac{1}{\epsilon })$, where $\epsilon$ is the desired precision in the output^24^. Dutta et al. propose a 7-qubit quantum circuit design for solving a 3-variable linear regression problem and simulate it on the Qiskit simulator^25^. Zhang et al. propose a hybrid approach for linear regression that utilizes both discrete and continuous quantum variables^26^. Date proposes the quantum discriminator, which is a quantum model for supervised learning^27^.

Adiabatic quantum computers have also been used to address machine learning problems in limited capacity. Foster et al. explore the use of D-Wave quantum computers for statistics^28^. Djidjev et al. use the D-Wave 2$\times$ quantum annealer for combinatorial optimization^29^. Borle et al. present a quantum annealing approach for the linear least squares problem^30^. Chang et al. propose a quantum annealing approach for solving polynomial systems of equations using least squares^31^. Chang et al. present a method for solving polynomial equations using quantum annealing and discuss its application to linear regression^32^. Neven et al. train a binary classifier with the quantum adiabatic algorithm and show that it performs better than the state-of-the-art machine learning algorithm AdaBoost^33^. Adachi and Henderson use quantum annealing for training deep neural networks on the coarse-grained version of the MNIST dataset^34^. Date et al. propose a classical quantum hybrid appraoch for unsupervised probabilistic machine learning using Restricted Boltzmann Machines and Deep Belief Networks^6^. Arthur et al. propose an adiabatic quantum computing approach for training balanced k-means clustering models^35^. Date et al. propose QUBO formulations for training three machine learning models—linear regression, support vector machine and k-means clustering—on adiabatic quantum computers^36^.

While several quantum computing approaches have been proposed for linear regression, most of them leverage universal quantum computers and not adiabatic quantum computers. Moreover, they have not been empirically validated on real hardware to the best of our knowledge. In this work, we propose a quantum computing approach for linear regression that leverages adiabatic quantum computers, which are sought to be more scalable than universal quantum computers in the near future^37^. Furthermore, we empirically validate our approach on synthetically generated datasets.

## Linear regression

**Figure 1:**
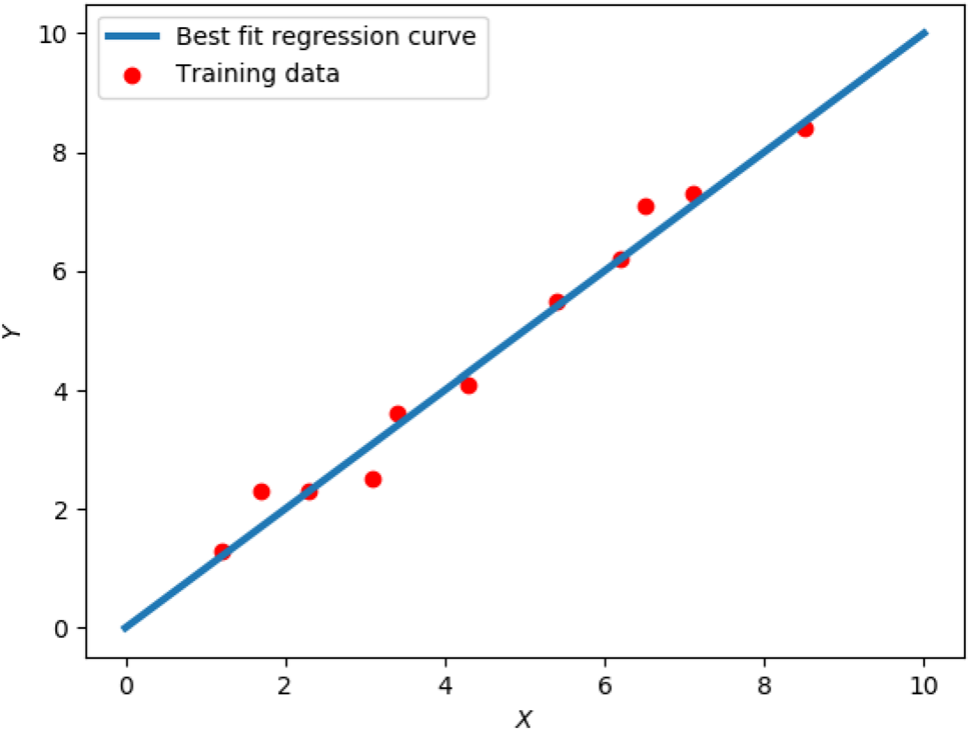
Linear regression. Red dots represent the training data for regression, and blue line represents the best fit for the given training data.

We use the following notation throughout this paper:

- ${\mathbb {R}}$: Set of real numbers
- ${\mathbb {B}}$: Set of binary numbers, i.e. ${\mathbb {B}} = \{0, 1\}$.
- ${\mathbb {N}}$: Set of natural numbers
- *X*: Augmented training dataset, usually $X \in {\mathbb {R}}^{N \times (d+1)}$, i.e. *X* contains *N* data points ($N \in {\mathbb {N}}$) along its rows, and each data point is a *d* dimensional row vector ($d \in {\mathbb {N}}$), augmented by unity, having a total length of $d + 1$.
- *Y*: Regression labels ($Y \in {\mathbb {R}}^N$), i.e. the dependant variable in linear regression.
- *w*: Regression weights to be learned, $w \in {\mathbb {R}}^{d + 1}$.

In Fig. 1, the red dots represent the regression training data and the blue line represents the best fit curve for the given training data. With reference to Fig. 1, the regression problem can be stated as follows:

1 $$\begin{aligned} \min _{w \in {\mathbb {R}}^{d+1}} \ E(w)&= || Xw - Y ||^2, \end{aligned}$$

where, *E*(*w*) is the Euclidean error function. The regression problem is one of the few machine learning problems which has an analytical solution, given by:

2 $$\begin{aligned} w = (X^T X)^{-1} X^T Y. \end{aligned}$$

If the inverse of $X^T X$ does not exist, the pseudo inverse is computed. The time complexity of linear regression is known to be ${\mathscr {O}}(N d^2)$.

## Formulation for adiabatic quantum computers

Adiabatic quantum computers are adept at approximately solving QUBO problems, which are NP-hard, and defined as:

3 $$\begin{aligned} \min _{z \in {\mathbb {B}}^M} z^T A z + z^T b, \end{aligned}$$

where, $z \in {\mathbb {B}}^M$ is the binary decision vector ($M \in {\mathbb {N}}$); $A \in {\mathbb {R}}^{M \times M}$ is the QUBO matrix; and, $b \in {\mathbb {R}}^M$ is the QUBO vector. In order to solve on adiabatic quantum computers, the regression problem needs to be converted into a QUBO problem. We start by rewriting Eq. (1) as follows:

4 $$\begin{aligned} \min _{w \in {\mathbb {R}}^{d+1}} E(w)&= w^T X^T X w - 2 w^T X^T Y + Y^T Y. \end{aligned}$$

Next, we introduce a precision vector $P = [p_1, p_2, \ldots , p_K]^T$, $K \in {\mathbb {N}}$. Concepts similar to the precision vector have been touched upon in the literature for encoding positive integers^30–32^. Each entry in *P* can be an integral power of 2, and can be both positive or negative. The precision vector must be sorted. For example, a precision vector could be: $P = \left[ -2, -1, -\frac{1}{2}, \frac{1}{2}, 1, 2, \right] ^T$. Next, we introduce *K* binary variables ${\hat{w}}_{ik}$ for each of the $d+1$ regression weights $w_i$ so that:

5 $$\begin{aligned} w_i = \sum _{k=1}^K p_k {\hat{w}}_{ik} \qquad \forall i = 1, 2, \ldots , d + 1, \end{aligned}$$

where, $p_k$ denotes the *k*th entry in the precision vector *P*. ${\hat{w}}_{ik}$ can be thought of as a binary decision variable that selects or ignores entries in *P* depending on whether its value is 1 or 0 respectively. With this formulation, we can have up to $2^K$ unique values for each $w_i$ when *P* contains only positive values for instance. However, if *P* contains negative values as well, then the number of unique attainable values for each $w_{i}$ might be less than $2^K$. For example, if $P = [-1, -\frac{1}{2}, \frac{1}{2}, 1]$, then only the following seven distinct values can be attained: $\{-\frac{3}{2}, -1, -\frac{1}{2}, 0, \frac{1}{2}, 1, \frac{3}{2}\}$. Next, we rewrite Eq. (5) in a matrix form as follows:

6 $$\begin{aligned} w&= {\mathscr {P}} {\hat{w}}, \end{aligned}$$

where, ${\mathscr {P}} = I_{d+1} \otimes P^T$ is the $(d+1) \times K(d+1)$ precision matrix obtained by taking the Kronecker product of identity matrix ($I_{d+1}$) with transpose of precision vector (*P*); and, ${\hat{w}} = [{\hat{w}}_{11}, \ldots , {\hat{w}}_{1K}, {\hat{w}}_{21}, \ldots , {\hat{w}}_{2K}, \ldots , {\hat{w}}_{(d+1)1}, \ldots , {\hat{w}}_{(d+1)K}]^T$ is the vector containing all $(d+1) K$ binary variables introduced in Eq. (5). These steps are taken for mathematical convenience. Now that we have expressed *w* in terms of binary variables ${\hat{w}}$ and precision matrix ${\mathscr {P}}$, we can substitute the value of *w* from Eq. (6) into Eq. (4), and convert the regression problem into a QUBO problem as follows:

7 $$\begin{aligned} \min _{{\hat{w}} \in {\mathbb {B}}^{(d+1)K}} E({\hat{w}})&= {\hat{w}}^T {\mathscr {P}}^T X^T X {\mathscr {P}} {\hat{w}} - 2 {\hat{w}}^T {\mathscr {P}}^T X^T Y. \end{aligned}$$

Note that we left out the last term ($Y^T Y$) from Eq. (4) because it is a constant scalar and does not affect the optimal solution of the unconstrained optimization problem. Also, note that Eq. (7) is identical to Eq. (3), with $M = (d+1) K$, $z = {\hat{w}}$, $A = {\mathscr {P}}^T X^T X {\mathscr {P}}$ and $b = - 2 {\mathscr {P}}^T X^T Y$. Thus, Eq. (7) is a QUBO problem and can be solved on adiabatic quantum computers.

## Analysis

### Theoretical analysis

The regression problem (Eq. 1) has ${\mathscr {O}}(N d)$ data (*X* and *Y*) and ${\mathscr {O}}(d)$ weights (*w*), which is the same for Eq. (7). While converting Eq. (1) to Eq. (7), we introduced *K* binary variables for each of the $d+1$ weights. So, we have ${\mathscr {O}}(d K)$ variables in Eq. (7), which translates to quadratic qubit footprint (${\mathscr {O}}(K^2 d^2)$) using the efficient embedding algorithm proposed by Date et al.^38^. Embedding is the process of mapping logical QUBO variables to qubits on the hardware, and is challenging because inter-qubit connectivity on the hardware is extremely limited.

As mentioned in Sect. 3, solving the regression problem (Eq. 1) takes ${\mathscr {O}}(N d^2)$ time. We analyze the time complexity of our approach in three parts: (i) Time taken to convert the regression problem into QUBO problem; (ii) Time taken to embed the QUBO problem onto the hardware; and (iii) Time taken to perform quantum annealing. From Eq. (7), we can infer that the conversion takes ${\mathscr {O}}(N d^2 K^2)$ time. Since we have ${\mathscr {O}}(dK)$ variables in the QUBO formulation, embedding can be done in ${\mathscr {O}}(d^2 K^2)$ time using the embedding algorithm proposed by Date et al.^38^. While the theoretical time complexity of quantum annealing to obtain an exact solution is known to be exponential (${\mathscr {O}}(e^{\sqrt{d}})$)^39^, a more realistic estimate of the running time can be made by using measures such as ST99 and ST99(OPT)^40^, which give the expected number of iterations to reach a certain level of optimality with $99\%$ certainty. Quantum annealing is known to perform well on problems where the energy barriers between local optima are tall and narrow because such an energy landscape is more conducive to quantum tunneling. In order to estimate ST99 and ST99(OPT) for our approach, details on specific instances of the regression problem are required. It remains out of the scope of this paper to estimate ST99 and ST99(OPT) for generic QUBO formulation of the regression problem.

Having said that, we would like to shed some light on the quantum annealing running times observed in practice. An adiabatic quantum computer can only accommodate finite-sized problems—for example, D-Wave 2000Q can accommodate problems having 64 or fewer binary variables requiring all-to-all connectivity^38^. For problems within this range, a constant annealing time and a constant number of repetitions seem to work well in practice. So, the total time to convert and solve a linear regression problem on adiabatic quantum computer would be ${\mathscr {O}}(N d^2 K^2)$.

It may seem that this running time is worse than its classical counterpart (${\mathscr {O}}(N d^2)$). But, the above analysis assumes that *K*, which is the length of the precision vector, is a variable. On classical computers, the precision is fixed, for example, 32-bit or 64-bit precision. We can analogously fix the precision for quantum computers, and treat *K* as a constant. The resulting qubit footprint would be ${\mathscr {O}}(d^2)$, and the time complexity would be ${\mathscr {O}}(N d^2)$, which is equivalent to the classical algorithm.

### Empirical analysis

#### Methodology and performance metrics

We test our quantum approach for regression using the D-Wave 2000Q adiabatic quantum computer and compare it to a classical approach using the Scikit-learn library in Python. The Scikit-learn library is widely used for machine learning tasks like linear regression, support vector machines, K-nearest neighbors, K-means clustering etc. We use two performance metrics for this comparison: (i) Regression error (Eq. 1); and, (ii) Total computation time. For D-Wave, the total computation time is comprised of the preprocessing time and the annealing time. The preprocessing time refers to converting the regression problem into QUBO problem and embedding it for the D-Wave hardware using our embedding algorithm from^38^. It must be noted that while working with D-Wave, there is a significant amount of time spent on sending a problem to the D-Wave servers, and receiving the solution back, which we refer to as network overheads. Although we report network overheads in Tables 2 and 3 for information purposes, we do not plot them in Figs. 3 and 4 and exclude them from our algorithm’s run time. This is because the network overheads are determined by factors like physical proximity of a user to D-Wave servers, network connectivity etc., which are neither in our control nor exclusive to our algorithm. In this paper, each quantum annealing operation is performed 1000 times and only the ground state solution is used. The value of 1000 was seen to yield the most reliable results based on trial and error for the experiments conducted in this paper.

#### Data generation

All data in this study, including the ground truth weights were synthetically generated, uniformly at random to curb any biases. We also injected noise into the data in order to compare robustness of both approaches and to emulate noisy nature of real world data. The precision vector *P* is constant across all our experiments, and the ground truth weights can be attained using the entries of *P*. We tried using benchmark datasets for regression like body fat, housing and pyrim^41^, but couldn’t generate any meaningful results because of the limitations imposed by the hardware architecture of the D-Wave 2000Q. These benchmark datasets require at least 16-bit precision and have several features. The D-Wave machine was too small to accommodate the QUBO problems that stem from these datasets. While it might be possible to deal with such benchmark data sets using D-Wave Hybrid Solver Service (HSS) or qbsolve, our objective in this paper it not to solve larger real-world or benchmark problems, but to objectively estimate the performance of the D-Wave quantum annealers for solving the linear regression problem.

#### Hardware configuration

Preprocessing for our quantum approach and entire classical approach were run on a machine with 3.6 GHz 8-core Intel i9 processor and 64 GB 2666 MHz DDR4 memory. The quantum approach also used the low-noise D-Wave 2000Q quantum computer, which had 2048 qubits and about 5600 inter-qubit connections.

#### Comparing regression error

**Table 1:** Comparing regression error.

Experimental runs where	Scikit-learn error	D-wave error
D-Wave fit the data ($68\%$ runs)	5.0597	5.1025
D-Wave did not fit the data ($32\%$ runs)	4.9340	16.1695
Overall	5.0195	8.6439

**Figure 2:**
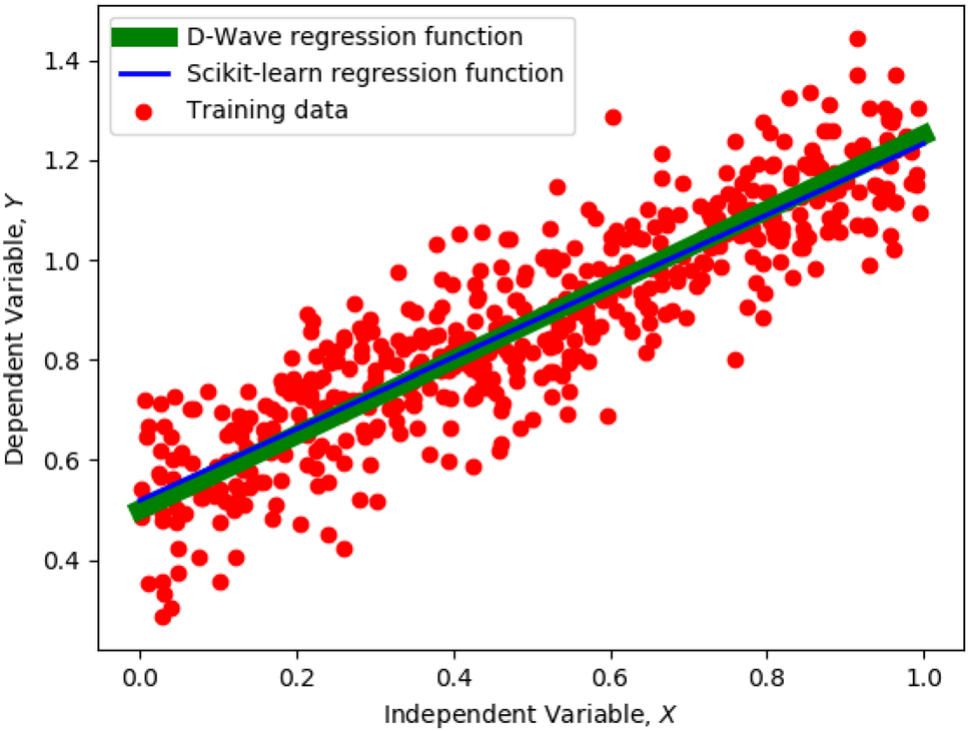
Comparison of regression curves fit by Scikit-learn (blue) and D-Wave (green) on synthetic data (red circles). X-axis shows the independent variable and Y-axis shows the dependent variable. Both curves closely resemble each other. We use a thicker green line and a thinner blue line for D-Wave and Scikit-learn respectively for the sake of clarity only—the two lines are very close to each other.

We compute regression error (Eq. 1) for our quantum approach using D-Wave 2000Q and compare it to the classical approach using Scikit-learn in Table 1. We report mean errors over 100 identical experimental runs to assess recovery rate of the D-Wave machine. The precision vector used for these runs was $P = [0.25, 0.5]$. We conducted experiments for all possible permutations and combinations of ground truth regression weights. Using the values in the precision vector, there are four unique values which the ground truth regression weights can have: 0, 0.25, 0.5, and 0.75. Using these values, we can have 16 different configurations of ground truth weights such as [0.25, 0.5], [0.5, 0.75], [0.25, 0.75] and so on. For each of these configurations, but ignoring the configurations where the weight is zero, we generated regression training data synthetically, added noise, fed this data to both classical and quantum approaches, and computed the regression error. We observe that the D-Wave approach fit the regression training data about $68\%$ of the time with a mean error of 5.1025. The mean Scikit-learn error for these runs was 5.0597.

While both errors were in the same ballpark, the Scikit-learn error was slightly lower than D-Wave because of the higher precision of the 64-bit classical computer. Within the 2-bit precision allowed by the precision vector *P*, D-Wave was able to find the best possible solution. An illustration of this is shown in Fig. 2, where regression data is shown by red dots, Scikit-learn function is shown by blue line and D-Wave function is shown by green line. The specific ground truth weights in Fig. 2 are [0.5, 0.75]. We see that the regression models trained on both Scikit-learn and D-Wave closely resemble each other, and are able to fit the data. In the case where D-Wave did not fit the regression data ($32\%$ of the time), mean D-Wave error was 16.1695. Mean Scikit-learn error for these runs was 4.9340. On an average, the Hamming distance (number of bit-flips) between the D-Wave solutions and the ground truth solutions was two across the four binary variables in these runs after application of post-processing routines to compensate for bit flips. The reason for this discrepancy is ingrained in the hardware of the D-Wave machine, which is known to produce faulty results when the embedded qubit chains break during quantum annealing^42^. Overall, mean errors for Scikit-learn and D-Wave were 4.9846 and 7.0421 respectively.

#### Scalability with number of datapoints (*N*)

**Table 2:** Scalability with number of datapoints (*N*).

Number of datapoints (*N*)	Scikit-learn time (ms)	D-wave preprocessing time (ms)	D-wave annealing time (ms)	D-wave compute time (preprocess + anneal) (ms)	D-wave network overheads (ms)
512	**0.7976** ± **0.0780**	0.2594 ± 0.0437	12.5151 ± 0.0186	**12.7744** ± **0.0461**	703.5815 ± 54.9066
1,024	**0.8274** ± **0.0957**	0.2543 ± 0.0261	12.5143 ± 0.0146	**12.7686** ± **0.0309**	703.0153 ± 34.5316
2,048	**0.8677** ± **0.0801**	0.2997 ± 0.0470	12.5152 ± 0.0105	**12.8149** ± **0.0481**	703.9943 ± 33.6994
4,096	**0.9259** ± **0.0890**	0.3284 ± 0.0337	12.5192 ± 0.0063	**12.8475** ± **0.0336**	689.5000 ± 35.5189
8,192	**1.0851** ± **0.0818**	0.3635 ± 0.1089	12.5205 ± 0.0036	**12.8840** ± **0.1088**	704.1441 ± 33.4093
16,384	**1.2458** ± **0.0895**	0.3041 ± 0.1913	12.5166 ± 0.0070	**12.8207** ± **0.1904**	716.4246 ± 45.7286
32,768	**1.6180** ± **0.0975**	0.4304 ± 0.2380	12.5129 ± 0.0079	**12.9433** ± **0.2368**	712.0551 ± 35.4758
65,536	**2.7692** ± **0.1485**	0.5584 ± 0.3751	12.5186 ± 0.0080	**13.0770** ± **0.3760**	718.3913 ± 40.5731
131,072	**4.8113** ± **0.2198**	1.1546 ± 0.6897	12.5149 ± 0.0112	**13.6695** ± **0.6906**	702.8292 ± 38.7911
262,144	**9.9080** ± **0.6120**	2.7862 ± 1.0094	12.5155 ± 0.0076	**15.3017** ± **1.0088**	711.5130 ± 37.2957
524,288	**19.5373** ± **1.0212**	5.1193 ± 0.3992	12.5166 ± 0.0030	**17.6358** ± **0.3983**	709.6294 ± 39.2782
1,048,576	**37.3581** ± **1.8984**	10.4900 ± 0.6307	12.5167 ± 0.0024	**23.0067** ± **0.6307**	707.4266 ± 38.3336
2,097,152	**73.6735** ± **3.4312**	27.0889 ± 1.3411	12.5175 ± 0.0025	**39.6064** ± **1.3413**	716.9348 ± 36.1262
4,194,304	**159.1724** ± **8.2130**	55.3273 ± 3.1763	12.5178 ± 0.0069	**67.8451** ± **3.1759**	713.8490 ± 52.9194
8,388,608	**328.2112** ± **13.0534**	103.6629 ± 4.5238	12.5170 ± 0.0036	**116.1799** ± **4.5245**	718.6187 ± 41.1655
16,777,216	**635.9468** ± **20.6696**	214.2371 ± 8.4610	12.5202 ± 0.0076	**226.7573** ± **8.4616**	710.6270 ± 32.7847

Significant values are in bold.

**Figure 3:**
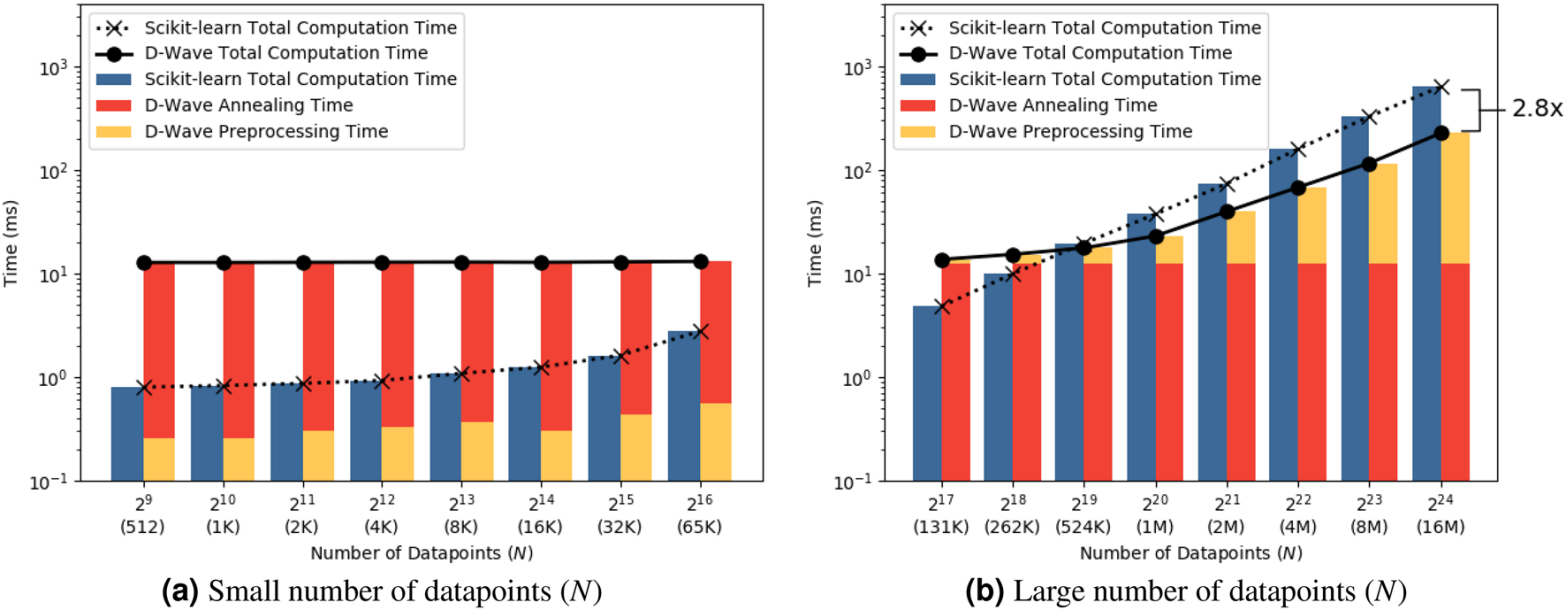
Scalability comparison of Scikit-learn regression (blue bars and dotted line) and D-Wave regression (yellow and red bars, and bold line). X-axis shows number of datapoints in the training set (*N*), ranging from $2^{9}$ (512) to $2^{24}$ (16 million) across both figures. Y-axis shows run time milliseconds on a logarithmic scale. In Fig. 3a, *N* varies from 512 to 65, 536. In Fig. 3b, *N* varies from 131, 072 to 16, 777, 216. We observe a $2.8 \times$ speedup using D-Wave on the 16 million case in Fig. 3b.

We perform a scalability study to determine how the run time of our quantum approach as well as the classical approach changes as the size of regression dataset increases from 512 datapoints to over 16 million datapoints. We report the mean and standard deviation across 60 runs in Table 2 and fix the number of features ($d+1$) at 2. The scalability results are presented in Fig. 3 where the logarithmic X-axis denotes number of datapoints (*N*), the logarithmic Y-axis denotes the time in milliseconds, the blue bars denote total Scikit-learn time, the yellow bars denote D-Wave preprocessing time, and the red bars denote D-Wave annealing time. We noticed that a constant annealing time of around 12.5 ms was sufficient to train the regression models using the quantum approach that had accuracies comparable to those of the classical approach for all our experimental runs. We observe that when number of datapoints is small ($N \le 262,144$), Scikit-learn performs faster than D-Wave. In this case, D-Wave compute time is dominated by annealing time and the preprocessing time is minimal. When the number of datapoints is large ($N \ge 524,288$), D-Wave performs faster than Scikit-learn. In this case, D-Wave compute time is dominated by the preprocessing time and the annealing time is minimal. The run times for the two approaches are comparable when *N* equals 524, 288 datapoints. When *N* equals 16, 777, 216, we observe that the quantum approach is $2.8\times$ faster than the classical approach. Furthermore, we also notice that D-Wave annealing time is essentially constant, and preprocessing time is always less than Scikit-learn time. This is attributed to efficiently converting regression problem into QUBO problem as described in this paper, and efficiently generating an embedding using our embedding algorithm^38^. The quantum approach seems to outperform the classical approach on larger datasets.

#### Scalability with number of features ($d+1$)

**Table 3:** Scalability with number of features ($d+1$).

Number of features ($d+1$)	Scikit-learn time (ms)	D-wave preprocessing time (ms)	D-wave annealing time (ms)	D-wave compute time (preprocess + anneal) (ms)	D-wave network overheads (ms)
2	**20.6123** ± **1.2042**	5.1378 ± 0.3802	12.5076 ± 0.0007	**17.6454** ± **0.3802**	706.1933 ± 84.0029
4	**30.6010** ± **1.2382**	13.7718 ± 0.9632	12.5247 ± 0.0009	**26.2965** ± **0.9632**	754.6531 ± 67.8583
6	**46.8912** ± **1.6430**	21.4310 ± 1.7784	12.5450 ± 0.0008	**33.9759** ± **1.7783**	756.5598 ± 66.0033
8	**68.4019** ± **3.9914**	28.9740 ± 2.0591	12.5659 ± 0.0006	**41.5398** ± **2.0590**	715.7661 ± 61.4712
10	**93.9764** ± **1.7518**	35.6321 ± 2.2202	12.5935 ± 0.0010	**48.2257** ± **2.2203**	761.3451 ± 60.9483
12	**118.1701** ± **2.0026**	42.5206 ± 2.6595	12.6092 ± 0.0012	**55.1297** ± **2.6596**	781.4883 ± 91.1495
14	**145.6177** ± **1.8870**	52.2676 ± 3.2121	12.6140 ± 0.0008	**64.8816** ± **3.2120**	844.3496 ± 107.1684
16	**175.5792** ± **2.4876**	60.6022 ± 3.7414	12.6195 ± 0.0010	**73.2217** ± **3.7415**	877.4846 ± 103.0307
18	**213.1724** ± **2.7332**	65.1949 ± 3.6451	12.6222 ± 0.0004	**77.8170** ± **3.6451**	791.8038 ± 105.9935
20	**236.3750** ± **4.6308**	74.9983 ± 3.9706	12.6212 ± 0.0004	**87.6194** ± **3.9706**	920.5470 ± 55.5933
22	**257.6503** ± **5.2920**	80.3314 ± 4.9133	12.6215 ± 0.0005	**92.9529** ± **4.9133**	779.6944 ± 90.0467
24	**281.3093** ± **4.0617**	83.6467 ± 3.6455	12.6211 ± 0.0007	**96.2678** ± **3.6457**	847.5243 ± 113.3988
26	**313.2982** ± **3.7929**	89.3653 ± 2.9930	12.6197 ± 0.0008	**101.9850** ± **2.9931**	804.1284 ± 91.5301
28	**343.8831** ± **4.3417**	98.9982 ± 4.5461	12.6174 ± 0.0010	**111.6156** ± **4.5462**	900.9338 ± 50.4548
30	**379.3123** ± **4.7932**	108.6154 ± 5.0667	12.6093 ± 0.0007	**121.2247** ± **5.0667**	1709.0392 ± 7766.58
32	**360.5327** ± **9.4234**	116.3282 ± 5.2455	12.5901 ± 0.0004	**128.9182** ± **5.2455**	706.8372 ± 73.4864

Significant values are in bold.

**Figure 4:**
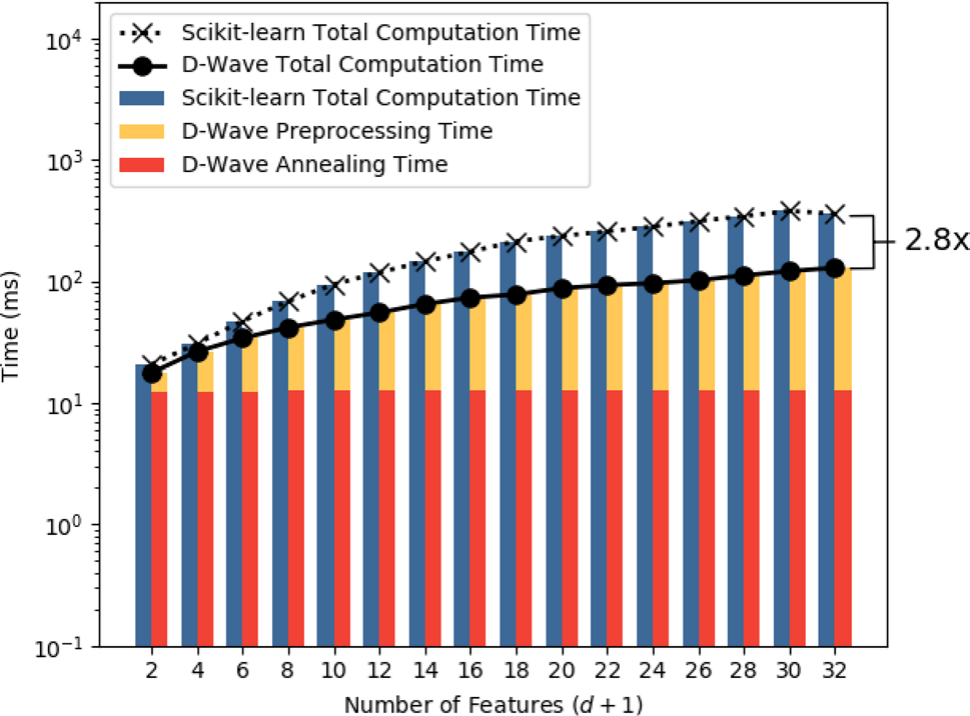
Scalability of Scikit-learn regression (blue bars and dotted line) and D-Wave regression (yellow and red bars, and bold line). X-axis shows number of features in the training set ($d+1$), ranging from 2 to 32. The Y-axis shows run time in milliseconds on a logarithmic scale. We observe a $2.8 \times$ speedup using D-Wave when $(d+1)$ equals 32.

We assess the scalability with respect to the number of features ($$d+1$$) as well. To eliminate the effect of number of datapoints, we fix *N* at 524, 288 datapoints because from Table 2 and Fig. 3, the run times of both quantum and classical approaches are comparable at this value. The results are presented in Table 3 and Fig. 4, where we vary the number of features ($$d+1$$) from 2 to 32. In Table 3, we report the mean and standard deviation over 60 runs for each experimental configuration. In Fig. 4, the X-axis shows number of features ($$d+1)$$, the logarithmic Y-axis shows run time in milliseconds, the blue bars denote total Scikit-learn times, the yellow bars denote D-Wave preprocessing times and the red bars denote D-Wave annealing times. In our quantum approach, by trial and error, we found that a constant annealing time of around 12.5 ms yielded regression models which had accuracies comparable to those of the classical approach for all our experimental runs. We observe that D-Wave performs faster than Scikit-learn for all values of $$d+1$$, and attains $$2.8\times $$ speedup when $$d+1$$ equals 32. We also observe that D-Wave run time is dominated by preprocessing time for almost all values of $$d+1$$, but is always less than Scikit-learn. This is attributed to efficient conversion of regression into QUBO as outlined in this paper, and use of our efficient embedding algorithm^38^. Lastly, we notice that the D-Wave annealing time is essentially constant across all values of $$d+1$$. As the number of features ($$d+1$$) increase, the quantum approach is seen to perform faster than the classical approach.

### Discussion

We first address why it is possible to scale *N* to over 16 million datapoints, but not possible to scale $d+1$ over 32 features. In Sect. 4, we show that the the qubit footprint (number of qubits used) of our formulation is ${\mathscr {O}}(d^2)$, and is independent of *N*, allowing us to scale *N* to over 16 million. We refrained from scaling *N* to larger values because we believe 16 million is a large enough value to convey the crux of this work—quantum computers can be valuable for solving machine learning problems like linear regression, especially on larger sized problems. We are limited to values of $d+1$ that are smaller than 32 because the qubit footprint depends on *d*. The size of the largest problem with all-to-all connectivity that can be accommodated on D-Wave 2000Q is 64, i.e. a QUBO problem having 64 variables. This is determined by the hardware architecture. Based on our formulation, the size of the regression QUBO problem is $(d+1) K$. So, problems for which $(d+1) K \le 64$ can be accommodated on the D-Wave machine. In our experimental runs, we fixed *K* as 2, and therefore, must have $(d+1) \le 32$. This limitation stems from the number of qubits and inter-qubit connectivity available on today’s quantum computers, and will improve in future quantum computers, which are sought to be bigger and more reliable than the current machines. For instance, the next generation D-Wave machines would have 5000 qubits and would support more inter-qubit connections^43,44^.

Secondly, we would like to reiterate that D-Wave was seen to produce accurate results about $68\%$ of the time during our empirical analysis, which is better than $50\%$ recovery rate previously observed by Chang et al.^32^. This result was found to be repeatable and could be attributed to hardware and software improvements made by D-Wave to their systems. During the remaining $32\%$ of the time, the inter-qubit connections on the hardware had a tendency to break, resulting in inferior solutions. This became increasingly prevalent on larger problems, which use large number of qubits. This hardware issue is expected to get better in the future as improved engineering solutions are deployed for building these machines.

Thirdly, we compared our adiabatic quantum linear regression approach to the Scikit-learn’s implementation of linear regression, which runs in ${\mathscr {O}}(Nd^2)$ time. Ideally, we would like for quantum algorithms to outperform the best classical algorithms, which in this case, runs in ${\mathscr {O}}(N d^{1.37})$. Having said that, we believe the novelty of our work lays in the extensive performance comparison of our quantum approach to its classical counterpart. Specifically, we are not aware of any study which compares an adiabatic quantum approach for linear regression to any classical approach as extensively as we have presented in this paper. Having said that, it is important to note that the $2.8\times$ speedup observed on larger-sized problems in our experiments depends on the specific implementations of the quantum and classical approaches. It should not be misunderstood as an absolute measure of quantum advantage or supremacy. We believe our results are a stepping stone in developing a more optimized approach to train linear regression models leveraging adiabatic quantum computers which can outperform the best classical approaches. Today’s quantum computers are still in their embryonic stages as compared to the classical computers, which have 70–80 years of research, development and optimizations behind them. In this light, we believe our results in this paper are extremely promising for the future of quantum machine learning. Specifically, with larger and more reliable quantum computers, we can expect the quantum approach to outperform the classical approach across all performance metrics.

Lastly, we would like to emphasize the algorithmic gains that could be realized by using our quantum approach for linear regression. In our empirical analysis, we observed that the quantum approach essentially had constant annealing time and the preprocessing time was always less than the run time of the classical approach. As a result, on smaller problems, the annealing time dominated the run time of the quantum approach and the overall time for the quantum approach was much worse than the classical approach. However, on larger problems, the annealing time stayed constant while the preprocessing time for the quantum approach was still lower than the run time for the classical approach. As a result, the overall run time for quantum approach was better than the classical approach. This observation can be attributed to the specific implementations of the Scikit-learn and Numpy functions. For embedding QUBO problems onto the D-Wave hardware, we tried using D-Wave’s embedding algorithm, but got significantly inferior results. All results in this paper use our embedding algorithm, which is described in^38^. Our quantum approach performed faster than the classical approach on increasingly large values of number of datapoints (*N*) as well as number of features (*d*). With quantum computers becoming less prone to errors in the future, it might be beneficial to use a quantum approach for linear regression, especially on larger problems.

## Conclusion

Training machine learning models for real world applications is time-intensive and can even take a few months in some cases. Generally, training a machine learning model is equivalent to solving an optimization problem over a well defined error function. Quantum computers are known to be good at (approximately) solving hard optimization problems and offer a compelling alternative for training machine learning models. In this paper, we propose an adiabatic quantum computing approach for training linear regression models, which is a statistical machine learning technique. We analyze our quantum approach theoretically, compare it to current classical approaches, and show that the time complexity for both these approaches is equivalent. Next, we test our quantum approach using the D-Wave 2000Q adiabatic quantum computer and compare it to a classical approach using the Scikit-learn library in Python. We demonstrate that the quantum approach performs at par with the classical approach on the regression error metric, and attains $2.8 \times$ speedup over the classical approach on larger (synthetically generated) datasets.

Continuing along this line of research, we would like to test our approach on real world datasets that can be accommodated on today’s quantum computers. We would also like to extend our quantum approach to variants of linear regression that use kernel methods. Finally, we would like to explore the use of quantum computers for training other machine learning models like Support Vector Machines (SVM), Deep Neural Networks (DNN), Generative Adversarial Networks (GAN) etc.

## Data Availability

The data that support the findings of this study are available from the corresponding author upon reasonable request.
